# Everything Hertz: methodological issues in short-term frequency-domain HRV

**DOI:** 10.3389/fphys.2014.00177

**Published:** 2014-05-07

**Authors:** James A. J. Heathers

**Affiliations:** Psychophysiology Group, Department of Psychology, University of SydneySydney, NSW, Australia

**Keywords:** heart rate variability, autonomic nervous system, sympatho-vagal balance, sympathetic nervous system, parasympathetic nervous system

## Abstract

Frequency analysis of the electrocardiographic RR interval is a common method of quantifying autonomic outflow by measuring the beat-to-beat modulation of the heart (heart rate variability; HRV). This review identifies a series of problems with the methods of doing so—the interpretation of low-frequency spectral power, the multiple use of equivalent normalized low frequency (LFnu), high frequency (HFnu) and ratio (LF/HF) terms, and the lack of control over extraneous variables, and reviews research in the calendar year 2012 to determine their prevalence and severity. Results support the mathematical equivalency of ratio units across studies, a reliance on those variables to explain autonomic outflow, and insufficient control of critical experimental variables. Research measurement of HRV has a substantial need for general methodological improvement.

## Introduction

**Figure 1:**
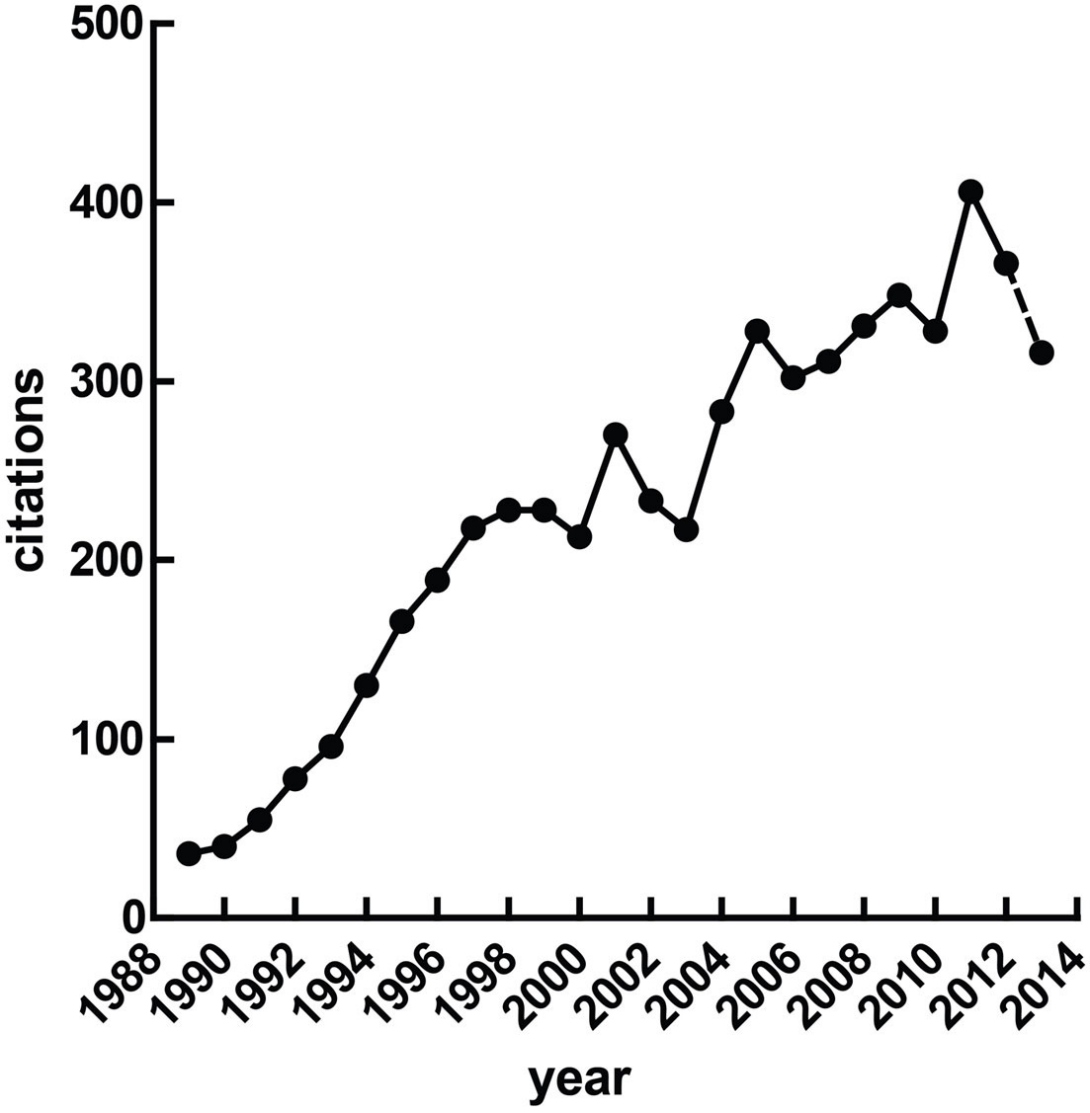
**Published research with “heart rate variability” in the title**. At the time of writing, the value for 2013 was extrapolated from the publications Jan 1st through April 30th.

Heart rate variability (HRV), the fluctuation of instantaneous heart period over time, is a correlate of cardiac autonomic regulation. HRV techniques have been applied in a broad range of contexts—they have been used to predict mortality after myocardial infarction (Buccelletti et al., 2009), as a correlate of stress (Berntson and Cacioppo, 2007) and psychopathology, to stratify attention (Mulder and Mulder, 1981), and have been incorporated into biobehavioral models of self-regulation (Porges, 1995; Thayer and Lane, 2000). The idea that reliable measurement of autonomic state may be obtained cheaply and non-invasively is obviously appealing. Figure 1 illustrates a growing interest in HRV methods over time, a trend which seem likely to continue given the increasing access to heart rate data through recent technological advances—heart rate has recently been accurately calculated via smartphone (Heathers, 2013), microwave (Suzuki et al., 2008) and induction-powered indwelling device (Riistama et al., 2007).

Short recordings of HRV (i.e., less than 1 h) typically show two primary patterns of oscillation which are separated into frequency bands from ≈7 to 25 s (0.04–0.15 Hz; low frequency, or LF) and 2.5 to ≈7 s (0.15–0.4 Hz; high frequency, or HF)—lower frequencies than LF are generally not meaningful over the short term. LF and HF frequency bands are widely used to quantify parasympathetic and sympathetic regulation (Akselrod et al., 1981) and their interaction (Malliani et al., 1991).

As ease of access to HRV increases, establishing and maintaining correct methodology is important—redundant methodology may delay treatment, obscure valuable underlying effects, provoke Type I or II errors, and most importantly, potentially delegitimize both alternative useful results and the utility of HRV in general. However, the internal and external consistency of the methods used have received comparatively less research interest than the understanding of the autonomic, cardiac and circulatory which creates those methods (Billman, 2011).

Thus, this paper presents a focused review of HRV methodology in the frequency domain which serves two purposes. Firstly, to raise several inter-connected points concerning the collection, interpretation and interrelationship of frequency-domain HRV variables, and external factors which may influence their recording. While there are many unsettled questions concerning meaning and calculation frequency domain HRV, the points raised here are generally not in dispute—they are either derived from a strong base of evidence, or are defined mathematically.

Secondly, to formally outline the awareness of these methodological points with reference to a large convenience sample of work using HRV methods. This sample is drawn from the most recent complete calendar year (2012) at the time of writing.

### The interpretation of LF power

All measures of HRV are necessarily complex as heart period over time is variously affected by multiple autonomic outflows, the modulation of those outflows at the sinoatrial node, their pacemaker response and competition, and the dynamic regulation of the vasculature, as well as endocrine, endothelial and mechanical factors. These interactions are further complicated by the realization that the individual mechanisms which may influence heart period are themselves incompletely understood. Examples include the source of cardiorespiratory coupling via either a central oscillator or the baroreflex (Eckberg, 2009; Karemaker, 2009), the mechanism behind periodic changes in blood pressure (Julien, 2006), the intrinsic meaning or function of respiratory sinus arrhythmia (Hayano et al., 1996; Tzeng et al., 2009; Ben-Tal et al., 2012; Elstad, 2012).

Irrespective of this, the power spectral density of high frequency HRV is strongly associated with cardiovagal activity (Akselrod et al., 1981; Kamath and Fallen, 1993; Malik, 1996). Respiratory variation observed in heart period is linearly related to parasympathetic control of heart rate (Katona and Jih, 1975), and its modulation forms the theoretical center of most HRV analysis. However, it should be noted that HF HRV is not abolished by vagotomy (Tzeng et al., 2005, 2007), and shows a complex and only somewhat dose-dependent relationship with muscarinic blockade (Picard et al., 2009).

Alternatively, the debate over the characterization, meaning and utility of LF HRV is ongoing issue (Akselrod et al., 1981; Porges and Byrne, 1992; Hopf et al., 1995; Introna et al., 1995; Sleight et al., 1995; Eckberg, 1997; Grasso et al., 1997; Malliani et al., 1998; Sleight and Bernardi, 1998; Houle and Billman, 1999; Notarius et al., 1999; Notarius and Floras, 2001; Elghozi and Julien, 2007; Billman, 2011, 2013; Goldstein et al., 2011; Pagani et al., 2012; Reyes del Paso et al., 2013).

To fully describe the physiology involved above is beyond the scope of this review. Within the present context, we may confine ourselves to addressing one common claim about frequency analysis—the involvement of the SNS in vasomotor control (Julien, 2006), and the strong relationship between the baroreflex and LF power (Goldstein et al., 2011) has occasionally been extrapolated to the position that LF power is proportional to cardiac sympathetic nerve activity. The direct evidence against this claim is strong even if confined to just non-invasive or minimally invasive studies in humans.

For instance, beta-adrenergic antagonists have shown divergent effects on LF power. Jokkel et al. (1995), for instance, report an approximate doubling of LF power in response to total beta-adrenergic blockade with propanolol (a non-selective β-blocker). A modest increase in LF power (Chiladakis et al., 2004) or no difference to baseline (Taylor et al., 1998) have been reported subsequent to treatment with atenolol (a β_1_-antagonist).

Likewise, cardiac 6-[^18^F] fluorodopamine imaging in humans (Goldstein et al., 1990, 1993), which radiolabels catecholamine storage vesicles, has repeatedly shown no relationship between radioactivity subsequent to cardiac sympathetic activity and LF-HRV power (Alvarenga et al., 2006; Moak et al., 2007; Rahman et al., 2011). Likewise, there are dissociations between other measurements of SNS via impedance cardiograph (Goedhart et al., 2008), salivary alpha-amylase (Nater et al., 2007; Kobayashi et al., 2012), circulating epinephrine/norepinephrine (Sloan et al., 1996), and muscle sympathetic nerve activity (Grassi and Esler, 1999). This evidence has been recently covered at length (Goldstein et al., 2011; Reyes del Paso et al., 2013).

The connection between LF power and sympathetic activity, while frequently cited as representative of (Pagani et al., 1984, 1986), is a misrepresentation of the initial claim that *normalized* LF power is representative of relative sympathetic power as a measure of sympathovagal balance. This, and related theory, is dealt with below.

### The LF/HF ratio

The ratio of low-frequency power to high-frequency power (LF/HF ratio), as popularized by (Pagani et al., 1984, 1986), is commonly used as a measure of sympathovagal balance—the putative balance between the mutually opposing branches of the autonomic nervous system. While widely used, this approach has been criticized on a number of grounds. The disconnection between this understanding of short-term spectral power within the heart series and the known physiology related to that power (Eckberg, 1997; Goldstein et al., 2011; Billman, 2013), and the response to those criticisms (Malliani et al., 1998; Pagani et al., 2012), have been covered in detail. As above, much of this is a natural extension of the argument that the numerator (i.e., LF power) reflects sympathetic outflow poorly, if at all.

From a methodological perspective, however, it is most concerning that there may be no mathematical basis on which to compare LF and HF power. Values of HRV are typically *internally consistent*, in that changes within a frequency band on individual sequential measurements may be directional or proportional. That is to say, it is meaningful that an individual under acute stress experiences a reduction in HF power from baseline, and that additive stress provokes additive change. However, those changes have less bearing on other measured quantities (i.e., a loss of HF power is compared to a loss of LF power; a loss of HF power between individuals, etc.). This is subsequent to considerations such that (a) fluctuations in HRV should more correctly be considered fluctuations in the *modulation* of autonomic tone, not a change in autonomic outflow (e.g., Katona et al., 1977), (b) the properties of interaction and competition between muscarinic and adrenergic outflow at the sinoatrial node are both non-linear (e.g., Levy, 1984) and mediated by neuropeptide co-transmitters (e.g., Revington and McCloskey, 1990), and c) changes in both low- and high- frequency power are mediated by both SNS and PNS (e.g., Taylor et al., 2001). This is often expressed simply by characterizing HRV as a qualitative, not quantitative, variable (Notarius and Floras, 2001; Billman, 2011).

However, the possibility of measuring sympathovagal balance in the manner above has been repeatedly classed as heuristic (Malliani et al., 1998; Sleight and Bernardi, 1998; Malliani, 2005). This position has a great deal of merit, as it is inevitable that complex or poorly understood phenomena will be demonstrably related to other dependent or independent variables in advance of our ability to explain why this is so. In other words, a metric may be *useful* well before it appears *meaningful*. One argument related to the above is the clear interrelationship during graded orthostatic tilt between (1) tilt angle, (2) sympathetic outflow, and (3) LF/HF ratio, the conclusion being that as all of these positively covary, then LF/HF ratio well describes, and is capable of predicting tilt angle (Montano et al., 1994). This may be the case, but only indicates an association between these factors, rather than a reason for their association.

**Figure 2:**
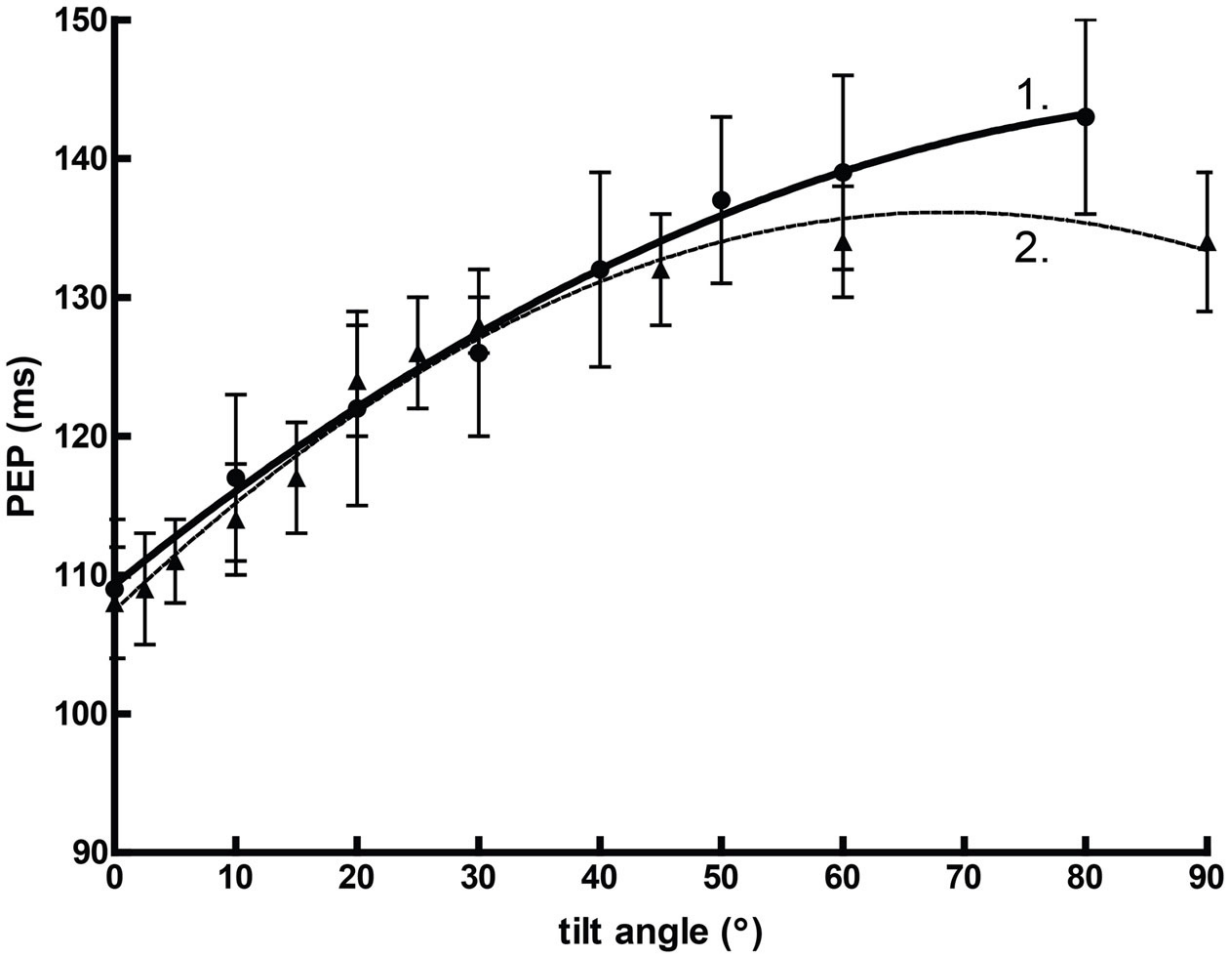
**The curvilinear relationship between pre-ejection period (PEP) and tilt angle during orthstatic stress**. Data from Chan et al. (2007) (1) and Stafford et al. (1970) (2).

Orthostasis provides an interesting comparative example. Figure 2 graphs mean pre-ejection period (PEP) against the angle of graded tilt with an overlaid quadratic regression (assuming that the relationship between cardiovascular response to tilt vs. angle is curvilinear). In this situation, the heuristic value of adjusted or unadjusted PEP is substantial, perhaps equivalent of some reports of spectral power (Bahjaoui-Bouhaddi et al., 2000), even without considering the individual regressions.

It seems very likely that predictions made in the manner of Malliani et al. (1997), where normalized units were successfully employed in a model to delineate posture, would be successful with PEP. Thus, as the following are clearly demonstrated:

1. there is a predictable, positive relationship between PEP and positive tilt
2. this relationship parallels an established positive relationship between SNS outflow and positive tilt (e.g., Chosy and Graham, 1965; Iwase et al., 1987)
  … we may draw a heuristic conclusion:
3. PEP is positively related to sympathetic outflow.

However, the normal relationship between PEP and SNS outflow is precisely the opposite. Sympathetic activity, as measured by circulating catecholamines (Chosy and Graham, 1965) or by MSNA (Iwase et al., 1987), increases reliably during orthostatic tilt. In other contexts, this might well accompany a decrease in PEP (Newlin and Levenson, 1979). However, our model here fails to account for the effects of preload—the initial stretching of the myocardium due to passive factors prior to the cardiac cycle—which increases proportionally with tilt angle independently of sympathetic drive (Stafford et al., 1970). Thus, a heuristic variable formed between two robust associations may be precisely predictive but ultimately misleading. This is precisely the criticism leveled by Grassi and Esler (1999); that LF/HF ratio fails to describe SNS outflow outside of the demonstration provided by changes in orthostasis.

Finally, the source of LF power is well characterized—LF power generally reflects the activity of the baroreflex in response to vasomotor tone. This is broadly accepted consequential to the classical demonstrations of the close correspondence between blood pressure waves and sympathetic modulation (Guyton and Harris, 1951), which are reflected in the heart period by the compensation of the baroreflex. This interpretation is not in dispute; a comprehensive summary is given in Berntson et al. (1997).

### The redundancy of normalized units and LF/HF ratio

Normalized HRV values (LFnu, HFnu) are calculated from the raw values of either short-term frequency band (LF or HF) divided by the total spectral power (typically LF + HF), with the value of this typically expressed as a percentage or decimal. These variables have a long history (e.g., Lombardi et al., 1987) in quantifying HRV, and have been used to quantify proportional sympathetic and parasympathetic activity respectively (e.g., Pagani et al., 1986). They are of particular interest in reviewing the available literature as they provide a degree of interpretability between studies, as proportional change between defined frequency bands can be seen as roughly equivalent regardless of the spectral method used. Unlike raw power, this allows direct comparison between frequency and autoregressive methods for calculating spectral power, between spectral power expressed as ms^2^ or bpm^2^, and between different algorithms for calculation, windowing methods, time periods, etc. These differences often result in baseline spectral values which are multiple orders of magnitude apart between studies (Sandercock, 2007).

However, the typical use of normalized units presents a series of significant redundancies. Firstly, LFnu and HFnu are trivially equivalent, as LFnu = 1-HFnu. This implies that calculations cannot be duplicated, as LFnu calculations are perfectly linearly related (i.e., computationally identical) to HFnu (Chemla et al., 2005). Reporting both values provides no additional information over reporting one, and change in one is identical to change in the other. In this manner, it is necessarily incorrect to refer to HFnu and LFnu as separate concepts. Instead, this model must describe a single autonomic continuum along which individual points represent the admixture of low and high frequency power. Furthermore, reporting calculations where only one normalized value is significant should be considered inconsistent.

There are exceptions to the above. Firstly, when normalized values are calculated from an expanded power spectrum; occasionally, Very Low Frequency (VLF; 0.003–0.04 Hz) may be included in the denominator of normalized units (i.e., LFnu = LF/VLF + LF + HF), likewise power about the HF cutoff (i.e., >0.4 Hz), or the total power of the observed spectrum (TP; 0–0.5 Hz) may be used as the denominator (i.e., LFnu = LF/TP; this is sometimes called LF%). However, in short recordings, the inclusion of these longer timescales is a significant problem as the contribution from very low frequencies is undersampled in the manner described below, and the Nyquist criterion prevents any meaningful contribution at frequencies higher than HF.

Secondly, when the autoregressive method is used to quantify spectral bands, often, the individual components identified for LF and HF bands sum to less than the measure of “total power”—the additive model minus the component at VLF. In this case, LFnu + HFnu will be less than 1, but most likely very close to it. As far as I am aware, there is no evidence to indicate that this establishes LFnu and HFnu as separate theoretical entities rather than measurement error. If the autoregressive model is a poor fit for the available data, then LFnu + HFnu may be significantly less than 1.

In addition, it is trivial to transform the LF/HF ratio as directly proportional to a normalized value of either spectral band (Burr, 2007):

$$\begin{array}{l} \;If\frac{LF}{HF}=\alpha ,\\ \;i.e.\;HF=\frac{LF}{\alpha }\\ \;then,\\ \;LFnu=\frac{LF}{LF+HF}\\ \;=\frac{LF}{LF+\frac{LF}{\alpha }}\\ \;=\frac{1}{1+\frac{1}{\alpha }}\\ \;i.e.\;LFnu=\frac{1}{1+{\left(\frac{LF}{HF}\right)}^{-1}}\\ \;and\;HFnu=\frac{1}{1+\left(\frac{LF}{HF}\right)} \end{array}$$

**Figure 3:**
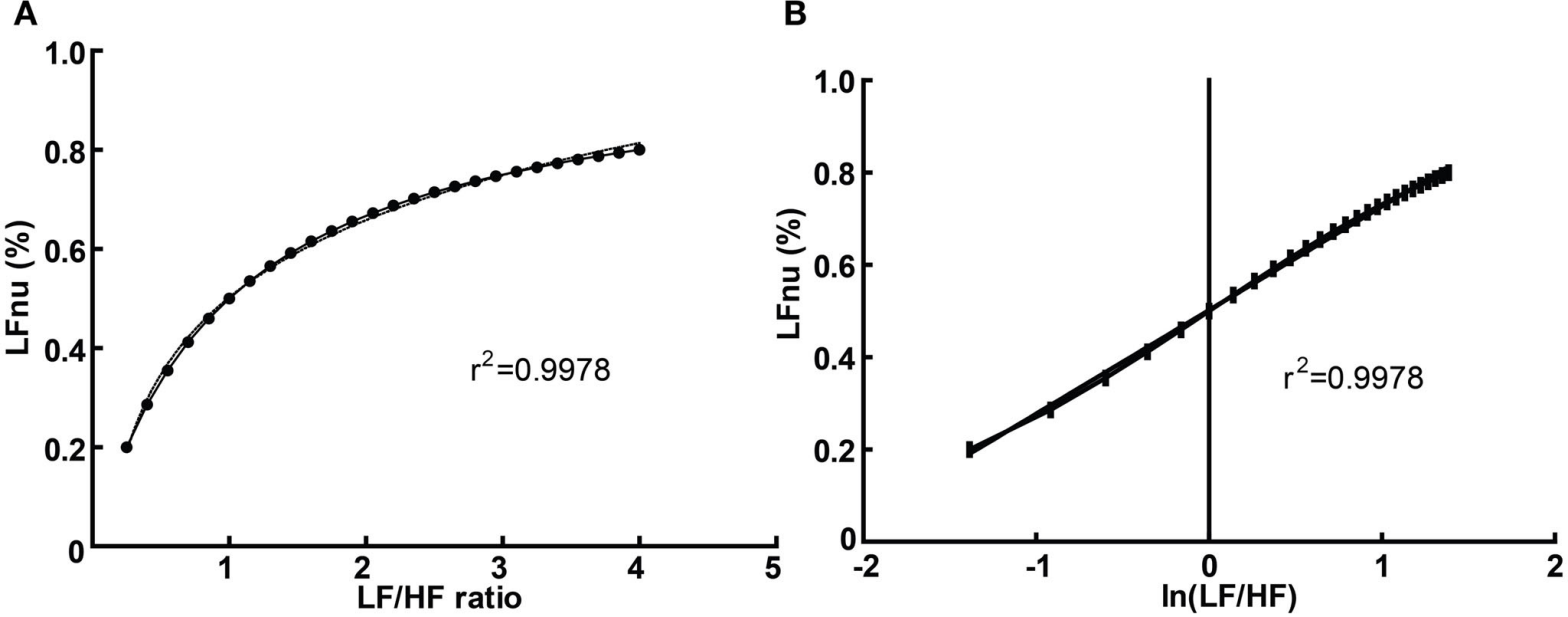
**The direct equivalence of normalized to ratio values with logarithmic regression (A), and of normalized to log-ratio values with linear regression (B)**. Values drawn from LFnu 0.2 to 0.8, *n* = 25.

Graphically, the function above is shown in Figure 3A—it is monotonically increasing at all positive non-zero values, non-linear, and well approximated by logarithmic regression over a typically observed range (*r*^2^ > 0.99). As the distribution of the LF/HF ratio is often positively skewed, it is frequently log-transformed to meet criteria of normality (e.g., Kobayashi et al., 2012). In this case, the non-linear relationship becomes significantly attenuated and very closely approximates linearity (Figure 3B)—thus a linear regression has an identical coefficient, constant term and *r*^2^-value.

In this manner, any given value of LFnu or HFnu has a directly equivalent LF/HF value. It should be emphasized that this is not a conceptual similarity but an equivalence at the level of definition—for example, an LF/HF ratio of 0.6 is precisely equivalent to LFnu = 37.5% or HFnu = 62.5%. Consequently, individual normalized values contain no more information than individual LF/HF ratio values, and on this basis it is unclear how “sympathetic balance” (LFnu) is mathematically different to “parasympathetic modulation” (HFnu) or how either is conceptually different to “sympathovagal balance” (LF/HF).

Similarly, due to the non-normal distribution of typical data, HRV variables are occasionally presented as median and interquartile range. As rank order is preserved in a monotonic increasing relationship, medians and inter-quartile values should remain direct transformations of each other, and statistical calculations on rank order should be identical between normalized and ratio values; a full description of this and other redundancies can be seen in Burr (2007).

**Figure 4:**
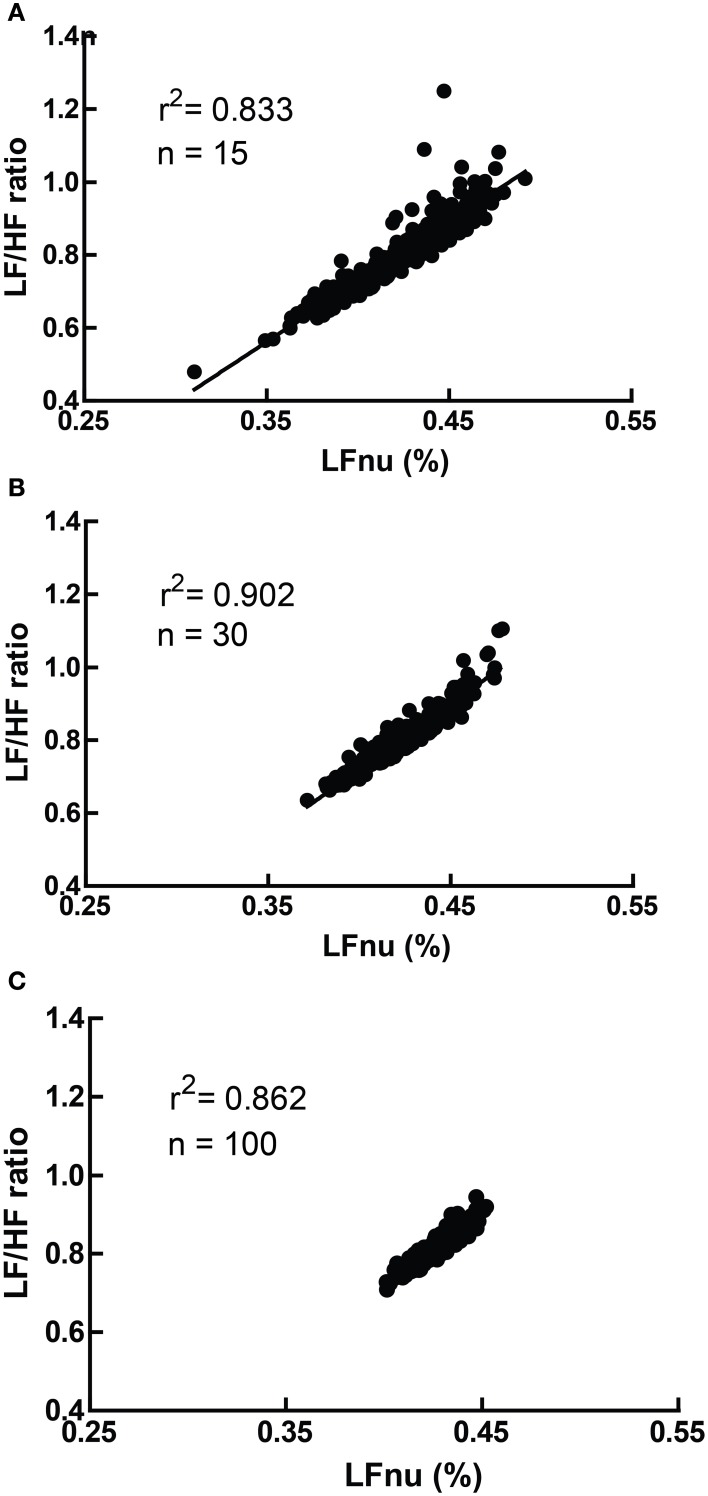
**A comparison of LFnu vs. LF/HF sample means, with (A) *n* = 15, (B) *n* = 30, (C) *n* = 100**. Points are means derived from approximately typical pseudorandom (Mersenne Twister) normally distributed values of LF ms^2^ (mean = 600, *SD* = 200) and HF ms^2^ (mean = 800, *SD* = 200). The values are distributed near the point of mean equivalence, LFnu = 0.429. r^2^-values range from 0.833 to 0.902.

However, due to the moderate non-linearity, mean (LF/HF) is not identical to mean (LFnu). This relationship is explored in Figure 4, where LFnu and LF/HF values from realistic artificial samples reveal a convergence toward the central value of LFnu with larger sample size, and a consistent predictive value between means. Thus, it is likely that statistical comparisons under standard parametric assumptions for LF nu and LF/HF would be similar without being identical.

### Interpreting normalized units in the absence of raw power

**Figure 5:**
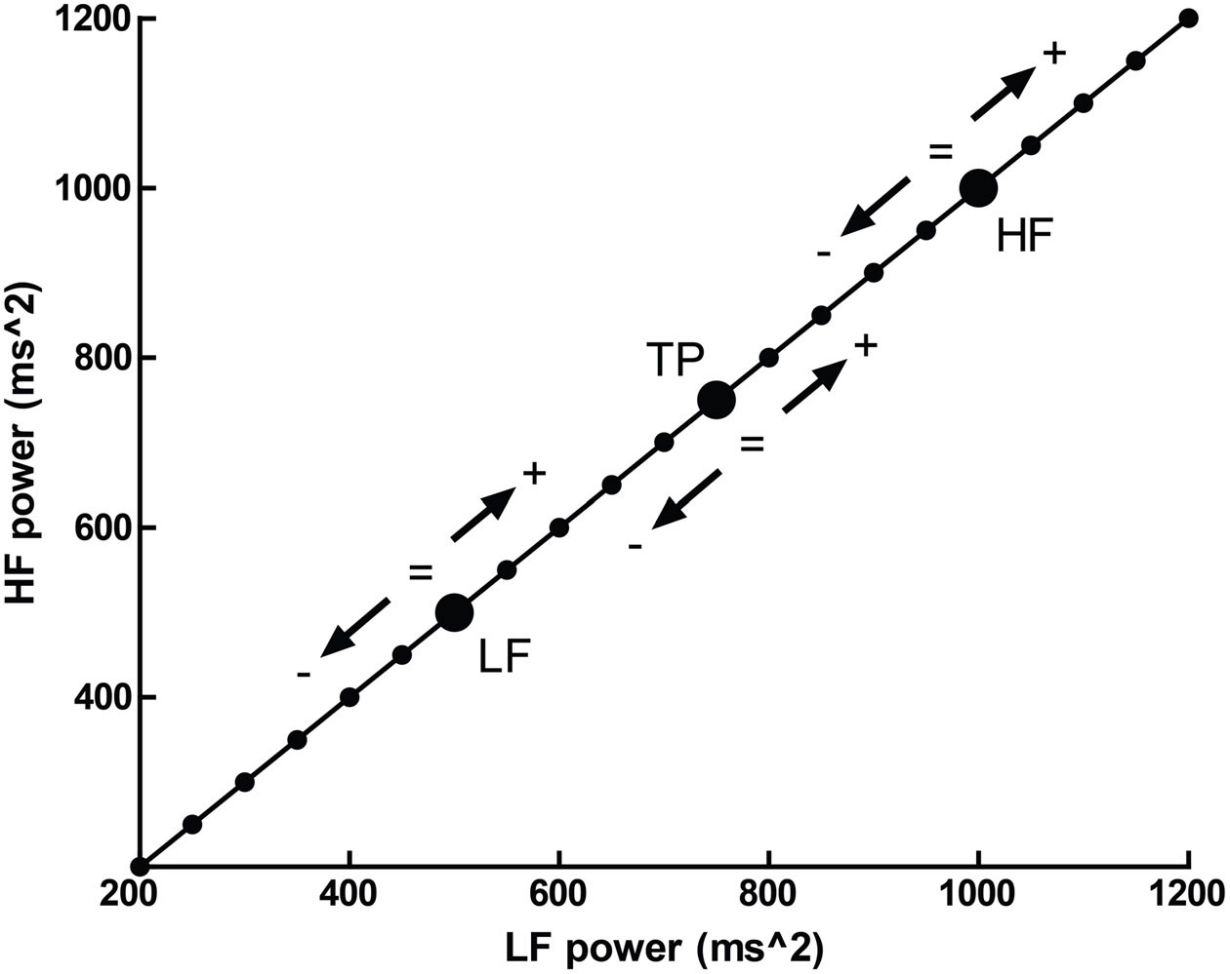
**The outcome of a hypothetical experiential effect—a participant with LFnu = 0.33 (LF ms^2^ = 500, HF ms^2^ = 1000) increases to LFnu = 0.5, which is defined by any point on the line of identity (i.e., LF ms^2^ = HF ms^2^)**. The arrows and operations on the line designate where values may be smaller, equal to, or greater for the corresponding spectral regions (LF, low frequency; HF, high frequency; and TP, total power).

Normalized units, which report frequency power proportional to the total observed power, possess an additional problem—that several different patterns of change in individual spectral bands may result in identical changes in proportion. This is illustrated in Figure 5, where a hypothetical participant with a baseline LFnu = 0.33 increases to LFnu = 0.5 after experimental intervention. This change in normalized units therefore represents not one possible change, but a continuum of possible changes which variously encompass (1) an increase, decrease or no change in (2) either total power, raw LF or raw HF power. Any point on the line of identity described in Figure 5 fulfills the criteria of LFnu = 0.5, but the individual points represent entirely different outcomes (Billman, 2013).

In other words, the reporting of HRV solely as a proportion directly obscures the underlying interpretation. It is precisely this form of interpretability which the seminal Task Force paper (Malik, 1996) sought to preserve within normalized values by recommending that research should always report both normalized and raw values for clarity.

**Figure 6:**
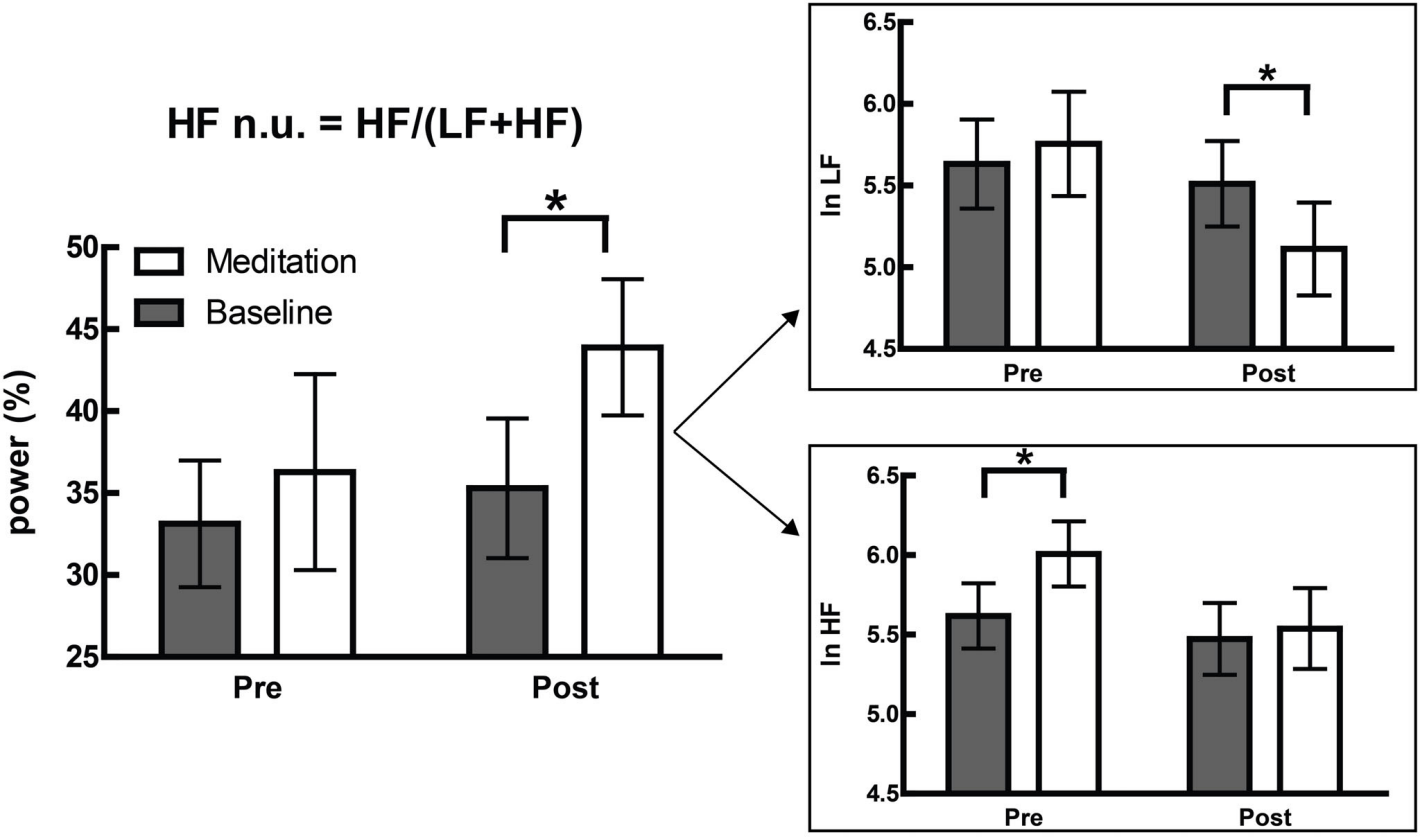
**Adapted from Krygier et al. (2013), Figure 1, with permission**. The devolution of the normalized results (top panel) into raw power (bottom panel) reveals two specific effects inconsistent with the overall interpretation of an alteration in autonomic balance. ^*^p < 0.05.

This is not merely a hypothetical scenario, and one of our recent papers illustrates this clearly (Krygier et al., 2013). In this study, comparisons of HRV metrics are drawn from a sample of meditators at rest and during Vipassana meditation, and both before and after an intense intervention—around 100 h of intensive training over 10 days. While the overall interaction was not significant, an intriguing and significant increase in HFnu was observed, as reported in previous research on similar forms of meditation (e.g., Sarang and Telles, 2006; Wu and Lo, 2008; An et al., 2010). A naïve characterization might be that a beneficial change representing an “increase in vagal tone” or a “favourable autonomic balance” was introduced by meditative training, but follow-up analyses revealed that normalized change was specifically mediated by (a) a profound increase in HRV at breathing frequency during meditation in untrained participants, and (b) a profound decrease in HRV at Mayer wave frequency during meditation when trained (Figure 6).

These changes precisely mirror the subjective reports of how meditative practice proceeds. Naïve practitioners of Vipassana, instructed to observe the breathing cycle rather than alter it, invariably “over-breathe,” which typically corresponds to an increased tidal volume and reduced respiratory rate. Within the lower portion of the HF spectra, this increases observed HF power (Hirsch and Bishop, 1981; Brown et al., 1993). However, this problem is mastered within a few days as participants practice the ability to passively observe normal respiratory cycles.

The above is a single unreplicated finding, and due to the nature of the task, breathing could not be consciously controlled (a potential confound, as breath has its own relationship to attention; see Vlemincx et al., 2012). Thus, while the above explanation is speculative, two points remain regardless: (1) the reference to individual frequency bands has greater explanatory power than the original naïve interpretation, especially considering changes in respiratory parameters, mood, attention, etc. are reliably predicted by spectral power in individual frequencies, and (2) the changes described within individual frequency bands may be entirely inconsistent with, and obscured by, the reporting of lone normalized HRV values.

### Time resolution of LF power

While an RR series does not consist entirely of cyclical processes (Peng et al., 1995), frequency analysis approximates the action of autonomic outflow to the heart by quantifying cyclical information present. In doing so, the number of times a cyclical frequency can be observed during an electrocardiographic recording varies linearly with the length of the recording, and inversely with the period of the frequency.

Consequently, HF HRV may be successfully recorded over periods of time as short as 60 s (Malik, 1996) as this gives adequate resolution to cycles within the heart period driven by respiratory sinus arrhythmia, typically around 0.25 Hz at rest. LF HRV requires a longer period in order for the spectral information to be reliably present. In short recordings, these frequencies may be insufficiently sampled—a signal at 0.04 Hz (i.e., with a period of 25 s) is observed 2.4 times per minute.

A heuristic rule which has been occasionally stated requires the sampling period to contain 10 complete cycles of the lowest observed frequency in order for the underlying information to be successfully approximated (Malik, 1996; Berntson et al., 1997) but there appears to be no analytical exposition of this. This has loosely translated into an accepted standard of a 5 min recording to measure short-term HRV, as a 5 min recording by this definition can resolve frequencies down to 0.033 Hz. Consequently, power from the LF spectrum down to 0.04 Hz is necessarily included in both normalized and LF/HF ratio calculations of HRV. Thus, both measurements should be taken over a minimum of 5 min.

### Extraneous variables to recording baseline heart period

Studies which measure variables that may be broadly affected by incidental day-to-day factors are usually carefully controlled. In human populations, research is often conducted specific to age group, experimental environment, time of day, medication status, environmental stimulants (i.e., caffeine or other methylxanthines), and so on. In longer studies or those requiring strenuous activity, standardized food and drink is provided. Studies in HRV are especially subject to these concerns—due to the autonomic innervation of the viscera, there are several instances where artifacts to short-term HRV measurement at rest may reliably arise from demographic variables, and the normal activities of daily living. Of course, controlling daily activity is not possible or even desirable in some patient groups, especially if long term monitoring is required (i.e., if measured over 24 h) but in laboratory or naturalistic experiments, it is ideal to observe potential changes in autonomic activity with as few confounding variables present as possible.

These variables are occasionally recognized; most research, for instance, is aware that HRV declines with age (O'Brien et al., 1986), is broadly affected by cardiovascular, vasoactive and psychotropic medication (e.g., beta-blockers; Sandrone et al., 1994), and is affected by both circadian rhythm (e.g., Massin et al., 2000) and wakefulness (Walker et al., 2009). Less frequently recognized is the finding that the autonomic innervation of the viscera means the consequences of feeding (i.e., the acute consumption of food and water, gastric distension and bladder filling) directly affect HRV.

Of these, the most attention has been paid to water consumption (May and Jordan, 2011) subsequent to the finding that patients with severe hypotension due to autonomic failure derived a significant reduction in symptoms from drinking water, and this subjective improvement was observed parallel to substantial increases in blood pressure (Jordan et al., 2000). A similar effect can be observed when the baroreflex loop is opened in sinoaortically denervated mice (McHugh et al., 2010).

In normal participants, the same presumed pressor effect takes place, and can be observed in muscle sympathetic outflow (Scott et al., 2001), but changes in blood pressure are immediately buffered by the efferent vagal baroreflex, and the immediate consequence is a moderate to large compensatory increase in heart period and HF-HRV. Healthy participants approximately double baseline HF-HRV, while the effects on HR are significant within 10 min after ingestion, peak at around 15–20 min and return to baseline by 45 min (Routledge et al., 2002). Recent work (Mendonca et al., 2013) has suggested that these effects only become negligent at VO2 maximum.

Eating and subsequent digestion have autonomic consequences which appear to be mediated both by gastric distention (Rossi et al., 1998) and by exposure to food-related stimuli (Nederkoorn et al., 2000). Mechanical and electrical stimuli to the stomach are both powerful hypotensive stimuli (Pozo et al., 1985), and this effect is abolished by vagotomy (Liu et al., 2004). In addition, the digestive process provokes vagal withdrawal as measured by HRV for at least 60 min after a meal (Lu et al., 1999), and increases sympathetic outflow to the skeletal muscles but not the heart (Fagius and Berne, 1994; Cox et al., 1995). Due to the relationship between the thermic effect of food and sympathetic outflow, this response is heavily affected by macronutrient composition (Welle et al., 1981; Schwartz et al., 1985).

Finally, bladder distension has been observed to provoke a robust series of pressor-mediated responses in humans (Fagius and Karhuvaara, 1989), where bladder distention predicts an increase in muscle sympathetic nerve outflow and blood pressure. Ben-Dror et al. (2012) subsequently delineated a linear rise in lnLF power with acute bladder filling in healthy controls drinking water. While this may have been confounded with the osmopressor effect (as above), a similar effect was observed using filling cystometry (i.e., causing bladder distension without drinking; Mehnert et al., 2009).

### Review parameters

In order to confirm both the nature and the extent of the problems outlined above, a substantial body of work is drawn from the recent HRV literature (i.e., from 2012). This allows the possibility of (a) sufficiently characterizing HRV research as it is presently performed with reference to the methodological issues raised, (b) confirming the presence and relevance of the mathematical relationships defined above, and (c) observing the extent of experimental controls currently employed.

## Methods

A non-systematic review was conducted: Google Scholar and PubMed databases were searched using the terms “heart rate variability” or “HRV” through either the title or abstract, with a date restriction of 01/01/12 through 31/12/12. Full text articles were obtained.

### Review process

Non-English language journals, 24 h studies (title/abstract: “Holter,” “24 hr”), animal (title/abstract: “mouse,” “rat,” “dog,” etc.), developmental (title/abstract: “neonatal,” “infant,” “child,” etc.), geriatric (title/abstract: “elderly,” “geriatric,” etc.), and conference abstract, qualitative or discussion papers (title/abstract: “editorial,” “conference,” “review,” etc.) were excluded, as were papers which were formally published in 2011 or 2013 (*n* = 293). The remaining papers (*n* = 573) were superficially reviewed to set initial criteria for inclusion.

### Selection criteria

#### Age

Pre-natal, infant, child and youth (mean age <18 years) samples, and elderly/geriatric samples (mean age > 65) were excluded.

#### Time period

Consistent recording for more than 1 h was not considered short-term and excluded. 24 h or Holter monitor studies were included only if a short-term period was additionally analyzed and reported to the daily record.

#### Descriptive work

Reviews, meta-analyses, position papers or commentaries, correspondence, etc. were excluded if descriptive of HRV phenomena instead of primary research, and included if they reported data from novel primary research.

#### Breathing

Paced breathing at speeds above 0.15 Hz was included. Breathing protocols slower than 0.15 Hz likely to affect the fundamental distribution of spectral power were excluded.

#### Healthy baseline condition

If plural baseline conditions were included within-subjects over one or multiple sessions, the first criteria reported—either by time, or if unclear, by listed order—was considered the baseline. If plural conditions were averaged to make a global value, this was considered equal to the total recorded time. If a baseline included plural subsequent measurement periods, i.e., two recordings of 3 min separated by task, then the first was used. Subsequent periods (i.e., “first 5 mins, second 5 mins”) were recorded as a single value if given otherwise the first period was used. Studies combining the averages of multiple time periods (i.e., the average of spectral values from two 3 min periods) were not recorded. Baselines immediately before surgery requiring general anesthesia were not considered resting, due to anticipatory anxiety. Multiple healthy groups from the same study were included if (a) listed separately at all points, and (b) were taken from baselines administered before random assignment into groups, or after assignment in benign circumstances. If sub-clinical groups from healthy populations were defined (i.e., “high normal” anxiety vs. “low normal” anxiety) then the low pathology group was used. Unless specifically stated as standing or supine, it was assumed that participants or patients were seated.

### Recorded information

#### LF power

The genesis of LF power provided was classified as being either (a) sympathetically mediated, (b) resulting from “both parasympathetic and sympathetic modulation,” (c) representing the gain of the baroreflex, or (d) other (parasympathetically mediated/not stated). Studies specifically measuring the LF response to graded tilt or postural change were taken as implying a relationship between LF and baroreflex outflow, as this is an orthostatic manipulation. If the basis of LF was derived from a reference without an explicit statement of what LF power was to represent, the interpretation within the reference was used according to the above criteria.

### Control of extraneous variables

#### Circadian

Circadian factors were considered controlled if both between and within subject comparisons were identical within a 24hr period, and confined to an hour or a time window of up to 4 h (i.e., “9 am to 1 pm” or “beginning in the early morning”).

#### Illness/medication

Work addressing serious, debilitating, psychiatric or other chronic illness, or any illness whose primary etiology was cardiovascular or circulatory, was included only if a control group was available, as baseline HRV level or collection/analysis technique may be affected. Non-life threatening illness treatable with standard pharmacotherapy (such as asthma) or post-treatment groups which did not require major pharmacotherapy or surgery (e.g., recovered phobics) were included. The exclusion or statistical control of any medication apart from the contraceptive pill or unscheduled analgesics (e.g., Paracetamol, Ibuprofen) was considered controlled.

#### Food/water

Meals were regarded as controlled either if participants were recorded during a fasted state, or if a standard meal was provided or prescribed for study inclusion, likewise water. A fasted state was assumed for participants measured at baseline before tilt-table testing. Water provided *ad libitum* was not considered controlled.

#### Bladder

Bladder emptying was only recorded if it was explicitly stated, as no pre-surgical population was included.

#### Content

With the exclusion criteria as above, the review proceeded pseudo-randomly (i.e., sequentially in alphabetical order by the surname of the first author) until 100 samples were recorded.

### Analysis

Comparisons between values were modeled respectively as the regressions HFnu = a/(b + c.(LF/HF)), LFnu = a/(b + c.(HF/LF)); all used the least-squares method and assumed initial conditions of any nominal constant = 1. The relationship between LFnu and HFnu was modeled by linear regression.

Relative standard errors (RSE; the standard error of the mean divided by the mean) were taken as measures of adjusted reliability for individual studies, and calculated from LF/HF ratios which were given in milliseconds squared, LFnu and HFnu values.

All calculations were performed in GraphPad Prism 5.

## Results

From *n* = 378 papers, *n* = 97 papers were accepted (*n* = 3 studies contained multiple baseline groups which met inclusion criteria), to give a total of *n* = 100 records of HRV at baseline. The list of these papers is included as supplementary material. If data was provided, participant age, sample size, HFnu mean and standard deviation (calculated from SEM if necessary), LFnu (likewise), or median and inter-quartile range were recorded separately. LF% and HF% were not recorded, as the inclusion of VLF power within short term calculations is problematic. All forms of spectral analysis (i.e., autoregressive method, FFT/DFT, Lomb-Scargle Periodogram, wavelet analysis etc.) were included as equivalent spectral analytical methods, as normalized units and/or LF/HF ratio were the recorded variables. The characterization of the acceptance/rejection criteria and use of spectral power is shown in Tables 1, 2.

**Table 1:** **Inclusion and exclusion criteria for reviewed studies**.

375	Reviewed	
278	Excluded	
	26	24 h or Holter monitor study	
	6	Animal	
	1	Duplicate record in database	
	11	Elderly, geriatric, or palliative sample	
	7	Elite or high level athletes	
	86	Exclusive to patient population	
	1	Incorrect calendar year (i.e., published 2013)	
	32	Infant, child or teenage sample	
	50	Letter, review, commentary, etc.	
	23	No resting baseline given	
	17	Non-linear, non-standard, etc. measures	
	13	Time domain measures only	
	5	Unavailable at the time of review	
97	Included	
	97	Met criteria	
	3	Multiple or duplicate usable records	

**Table 2:** **Reporting of raw vs. adjusted values, single vs. multiple normalized or ratio units**.

	**Reported raw values**	**No raw values**	
Single nu/Ratio unit	26	4	30
Multiple nu/Ratio units	26	18	44
	52	22	*n* = 74

Extraneous controls varied substantially between measures: of the 97 separate studies accepted, 81% controlled for medication or health status, 76% for nicotine use, 58% for time of recording, 45% controlled for food intake, 23% controlled for water intake, and 4% for micturition. Of the above *n* = 97 studies, 91 (94%) analyzed some version of LF power, and 74 (76%) reported at least one normalized or ratio unit measure. 50 papers specifically reported the LF/HF ratio: 13/50 (26%) reported log-corrected units and 37/50 (74%) reported uncorrected units.

The time periods used for HRV recording were primarily 5 min (*n* = 40; 41%), or 10 min (*n* = 20; 21%). Recording times under 5 min were uncommon (*n* = 13; 13%), with *n* = 10 (10%) of these using a measure of LF power.

**Figure 7:**
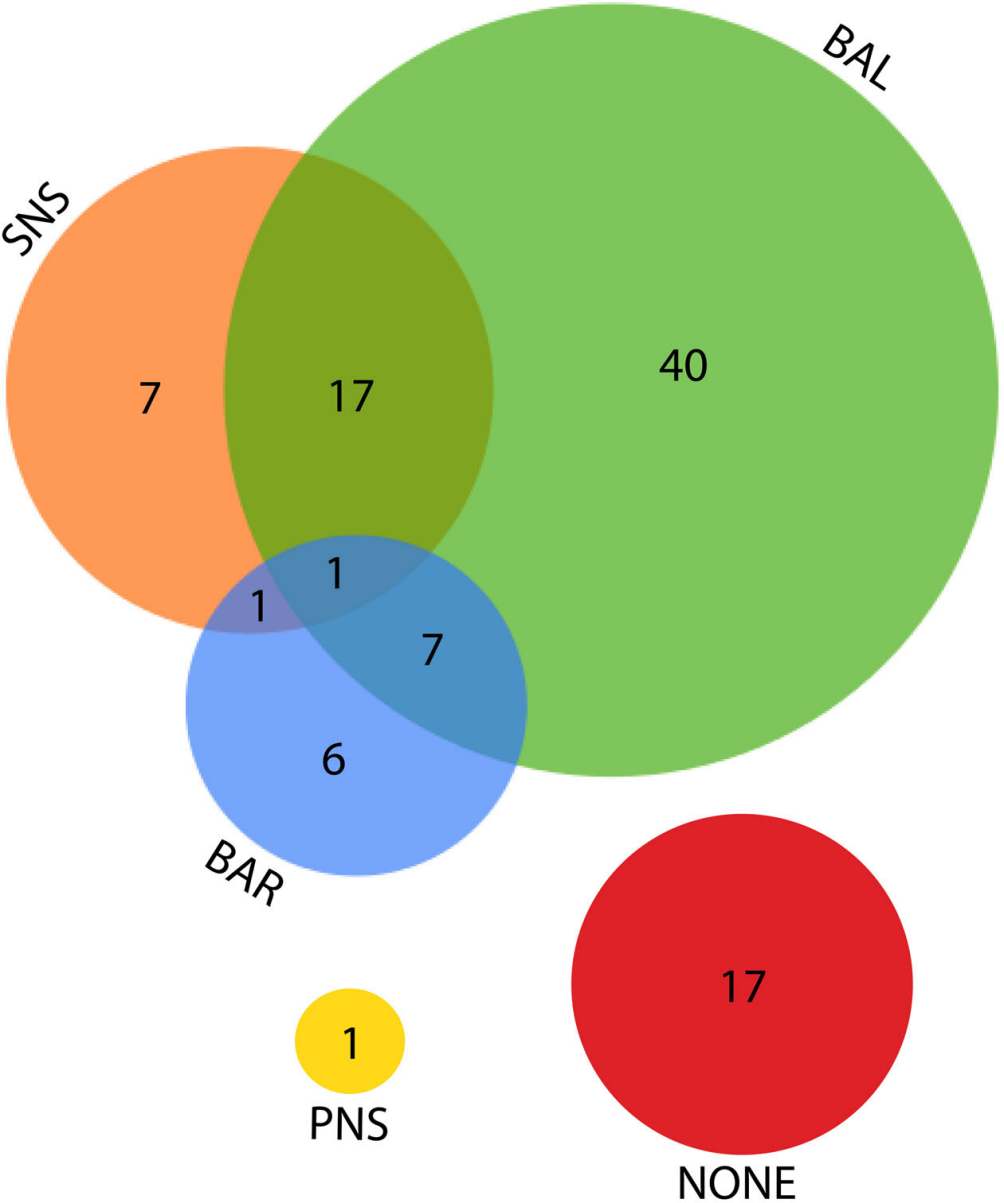
**Venn diagram for the various explanations given as the source of LF power**. SNS, sympathetic nervous system; BAL, a balance of parasympathetic and sympathetic influences; BAR, the activity of the baroreflex; PNS, parasympathetic nervous system.

**Figure 8:**
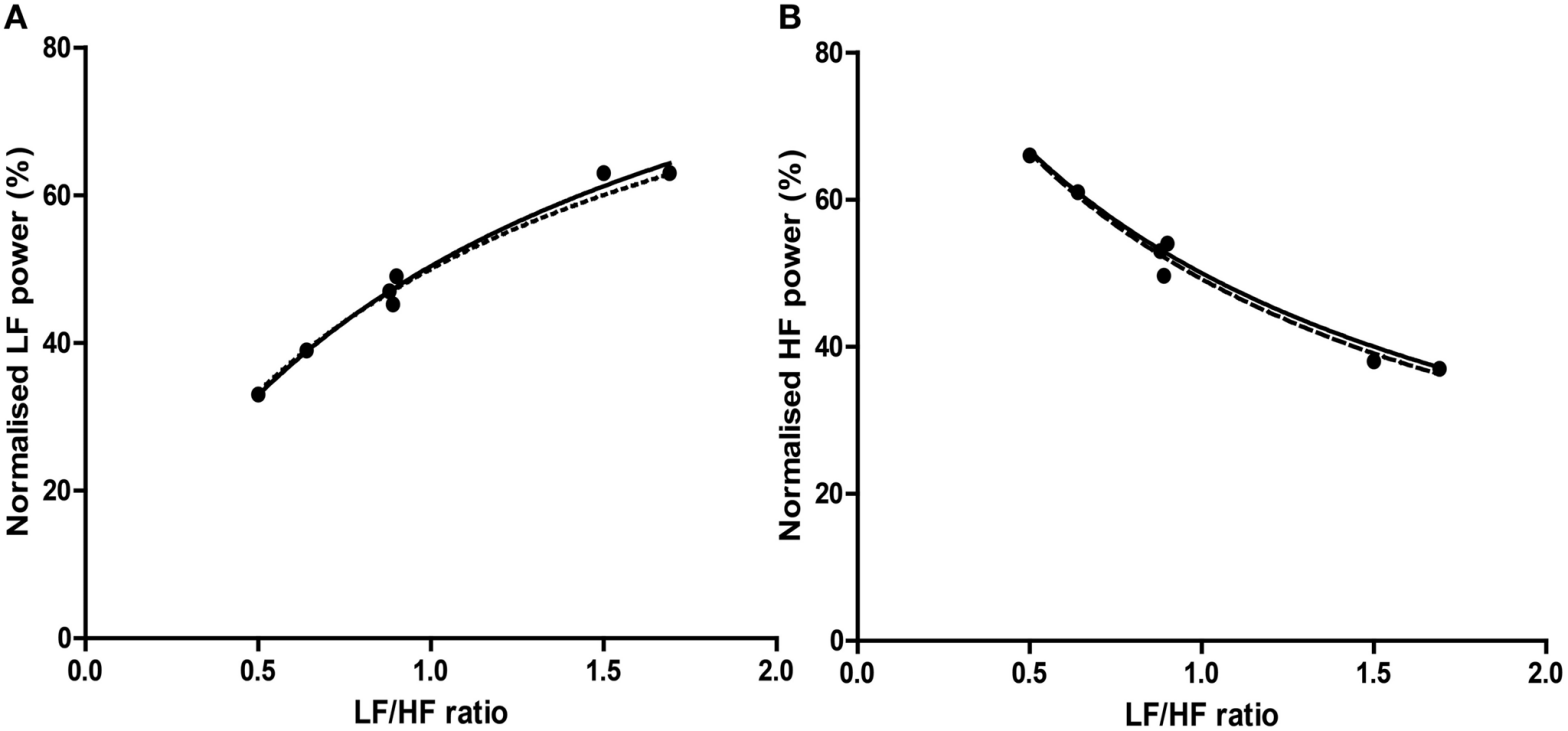
**The relationship of precisely equivalent values, i.e., median and interquartile range (IQR) between LFnu (A) or HFnu (B) and LF/HF ratio**. Regression estimates: **(A)** *a* = 1.00, *b* = 0.94, *c* = 1.06, *r*^2^ = 0.985, *n* = 7; **(B)** *a* = 1.00, *b* = 0.98, *c* = 1.05, *r*^2^ = 0.981, *n* = 7. Values were uncommon as few studies reported both normalized and LF/HF ratio values in median/IQR format. The dashed line represents the mathematical identity as previously defined.

**Figure 9:**
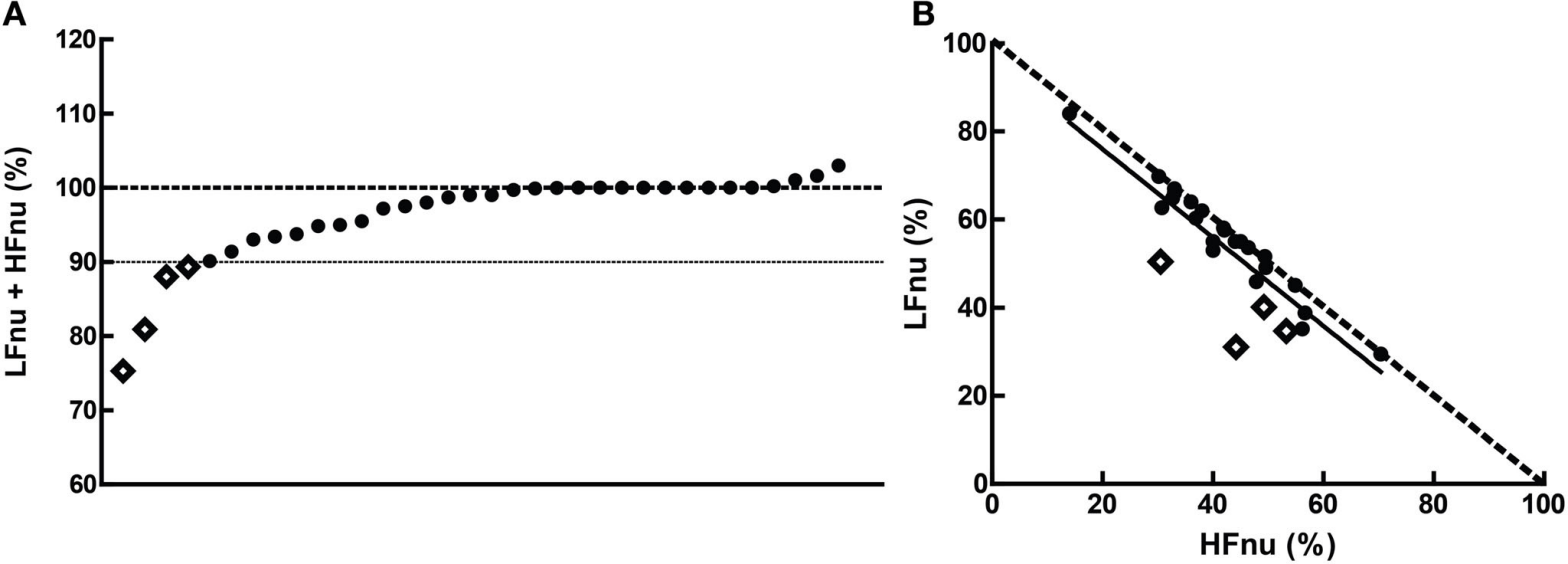
**The cumulative sums of the normalized components when (A) both were specified, and (B) their interrelationship (slope = −1.003 ± 0.1103, *r*^2^ = 0.761, *n* = 20)**. The dashed line in **(B)** represents the mathematical identity as previously defined. Points marked as diamonds are LFnu + HFnu <90%, and marked where relevant on Figure 10.

**Figure 10:**
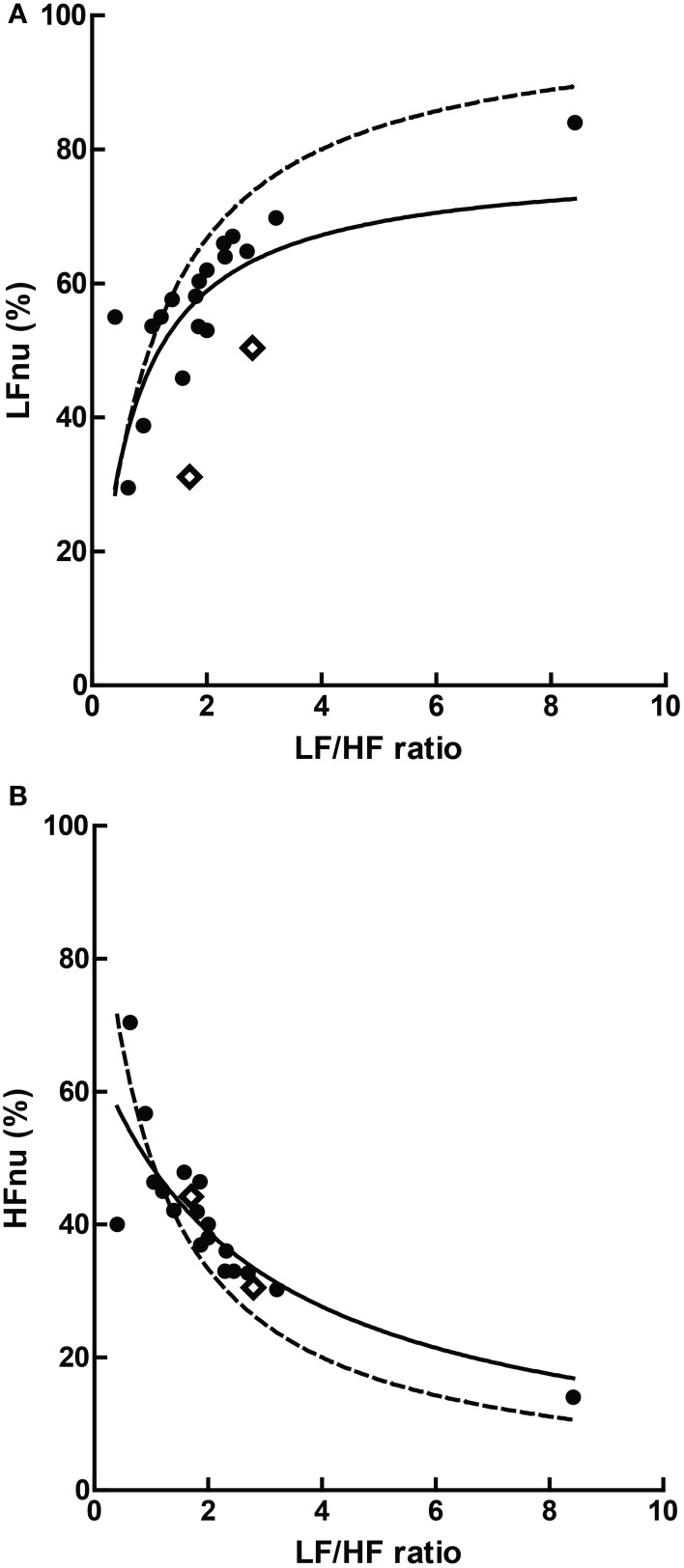
**The relationship of mean ratios to approximate mean normalized values, where (A) LF *r*^2^ = 0.363; (B) HF *r*^2^ = 0.685, *n* = 20**. The dashed lines represent the mathematical identities as previously defined.

**Figure 11:**
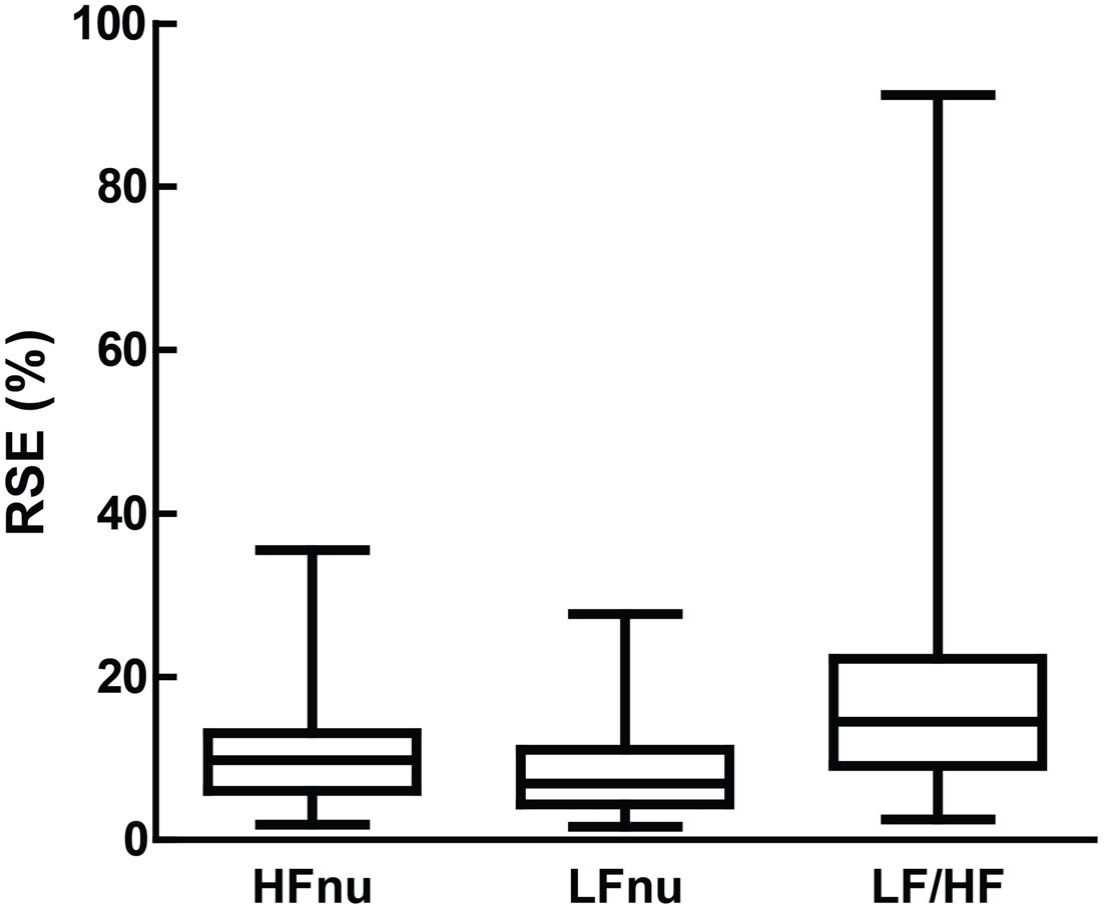
**Relative standard error (i.e., the standard error divided by the mean, which forms the sample-sized adjusted coefficient of variation) for LFnu (*n* = 29), HFnu (*n* = 30) and LF/HF (*n* = 30), shown here by median, interquartile range and min/max**.

Remaining figures are descriptive of the parameters of review; Figure 7 describes the primary interpretation given to ratio or normalized power. The number of points available for each individual comparison below is noted separately per figure. Figure 8 shows the means and relevant interquartile values assumed to be precisely equivalent due to equal rank order with the inverse-term regression relating LF/HF and nu units overlaid. Figure 9 describes the sum and interrelationship of normalized values assumed to be precisely equal to unity. The relationship between mean normalized units and mean LF/HF ratio is shown in Figure 10, and their precision is shown in Figure 11.

## Discussion

Overall, the use of frequency analysis over short-term heart rate recordings to characterize autonomic state or sympathovagal balance is problematic. Relevant research frequently truncates or fails to explain the source of HRV power. Commonly co-investigated variables are reported as separate concepts, but are mathematically redundant as predicted. This redundancy is precise between individual values and moderate between group means. Time periods employed for recording are generally sufficient. Confounding variables which have the potential to substantially alter between- and within-subject variance are infrequently controlled.

### Overall precision and experimental control

The control of extraneous factors affecting recording in participants is perhaps the most problematic of the results here, because it may irreparably affect the veracity of between-subjects experimental models. Of course, depending on the circumstances, it may not be possible or even desirable to control all the listed variables—for instance, patient populations must remain on medication, opportunistic recording at any time of day is necessary to observe an episodic phenomenon, etc. However, the fact remains that circadian rhythm, medication, health status, food, water and bladder filling all potentially possess the ability to modify the variance of a normative group, even if only problematic in a minority of participants. Some of these external factors (medication, health status, and nicotine use) are well controlled, but a minority of work controlled for gastric or bladder filling. The amount that this affects a normative sample of HRV needs to be determined experimentally.

For experimentation within subjects, the situation is a lot less clear. Obviously, if within-subject measurement involves an intervention over multiple recording periods in time, the potential contamination presents precisely as it would between subjects. However, if a task effect is being observed in sequential recording periods during the same experiment, the problem may be substantially reduced. That is, in the presence of a strong artifact, the absolute or proportional change in HRV in response to a drug, task, intervention etc. may occur reliably but simply from an altered baseline.

Say, for instance, that gastric activity subsequent to feeding increases LF spectral power in an experimental participant who is then subjected to social stress, which is also expected to increase LF power. If that power rises to a proportional level, i.e., rises by the same absolute or proportional amount that it otherwise would in the absence of feeding, then any potential source of error has been substantially ameliorated by the design.

The problem in this instance would be amplified if there was an interaction between the altered baseline and task. If the response is attenuated or amplified, i.e., there is an interaction between the task effect and the source of artifact, then the situation is concerning, doubly so if a small sample is being used. To a small sample with normative values (e.g., Nunan et al., 2010; *LF* = 519 ms^2^, *HF* = 657 ms^2^), a mean increase in HF ms^2^ subsequent to drinking (Routledge et al., 2002; HF +686 ms^2^) has the potential to destroy the fidelity of an entire measurement at baseline. If this change interacts with any given task-related effect, the sample quickly runs the risk of becoming uninterpretable.

### Normalized and LF/HF variables; co-reporting and equivalence

Co-reporting of equivalent ratio values is reasonably common, observed in over half (59%) of the studies which employed normalized units or LF/HF. The definition of these measures as redundant is borne out by the results. The argument might be made that this is not problematic, as HRV studies typically employ a range of time and frequency domain measures which are multicollinear. This is impossible to avoid, as most HRV methods some manner of apportioning a meaning to some quantity of the available variance in a heart period series, and cross from time to frequency domain readily as the integral of the total power spectrum is equal to the variance. These interrelationships are often very high—Massin et al. (1999) report, for example, that RMSSD, pNN50 and HF power are mutually correlated above 0.9.

However, there are several problems with this line of argument when applied to multiple ratio measures. It is generally accepted that multiple similar measures of HRV might be employed to the same end to address the same phenomenon may differ slightly—for example, in a group undergoing an experimental intervention, RMSSD may be significantly increased by task and HF power not. Were this the case, the result would be taken as equivocal support for a change in cardiac vagal modulation, and the difference in the result between the similar calculatory methods would be addressed. Alternatively, if LFnu was significant and LF/HF not, this might be interpreted as a change in relative sympathetic activity, but no change in sympathovagal balance. Secondly, other measures which may be closely correlated attempt to measure the same phenomenon but use entirely disparate methods. However, normalized and ratio values are mathematically, not theoretically, related. There is no equivalent transform which might imply, say, an RMSSD value from a value of HF spectral power.

Thirdly, it is unclear which of the ratio measures best represents the comparison they are both attempting to capture. For instance, normalized units are more likely to obey parametric assumptions, but LF/HF may exhibit significant skew and kurtosis (Kobayashi et al., 2012). This is directly confirmed in Figure 11, as the relative standard error of both normalized units is substantially lower than LF/HF. If the relationship is mathematically equivalent, and we also accept a degree of measurement error, how should we interpret an instance where one value is significant and the other not? Is it inconsistent to report both if one works? If a sample of LF/HF obeys standard parametric assumptions, should it still be log-transformed?

Lastly, it is by no means uncommon for normalized spectral bands and the LF/HF ratio to form the entirety of an analysis in an attempt to measure relative sympathetic and parasympathetic contributions, and their interrelationship. This is rarely the case with other interrelated variables.

### Normalized variables and unity

Overall, the predictions from the mathematical equivalence in the introduction were borne out—the curvilinear relationship between normalized and ratio figures were observed. In some comparisons, however, slight to significant departure can be seen contingent on normalized values adding to unity—this was the case for a minority of studies observed, with 4 of the 34 observed sums of LFnu and HFnu below 90%. These departures are reflected in Figure 9, and are marked as diamonds between all graphs for continuity.

There are multiple, non-mutually exclusive possibilities for this discontinuity which are not simply calculatory error. The first is the use of an alternative definition of adjusted LF power, i.e., LF/(total power). As the contribution from VLF power is usually significant, this may explain the larger error but not the preponderance of values from 95 to 99% of the sum LFnu + HFnu. The second is that there are small but significant contributions to spectral power above 0.4 Hz, which are included in total power but not included as part of the HF frequency band—this might explain the frequent values close to 1, but not the significant deviations from it. The third is confusion in the calculation of the autoregressive method between the dominant power components in each spectral band which frequently overlap into the other segments, and the power spectral density of all components but strictly within the defined power band.

This non-equivalence is responsible for the shift in the distribution that can be seen in Figure 9—the bulk of the points are distributed as expected, but lie rightwards of the line of definition, where the ratio value is slightly bigger than predicted. The regression of the data conforms to this.

It cannot be concluded precisely what this difference represents. The best case scenario for the use of normalized units would be that this difference is borne of the fact that the individual spectral powers retain some statistical independence, and describe a portion of the relevant variance in their spectral bands without absolute covariation.

The worst case scenario is that this is simply a calculatory curiosity which has no specific meaning, borne of the arbitrary distinction between the whole spectral element (say, the power under the peak which provides the bulk of LF power) and the truncated version (say, the PSD from precisely 0.04 to 0.15 Hz of all components). If the “true” sum of LFnu and HFnu is always 1, then their statistical equivalence is complete—in comparisons, the metrics they return for continuous or directional comparison (e.g., Spearman's r, Student's t) to other variables will differ by sign, and *F*-values not at all. If this is the case, then this error has previously allowed the precise equivalence of LFnu or HFnu to be partially obscured, and normalized/ratio units substantially obscured, by providing values which are somewhat divergent and giving the appearance of independence.

### Dispersion of ratio values

LF/HF ratio shows an obvious decrease in precision over either normalized variables (Figure 11). This is likely due to the volatility of the LF/HF ratio during normal sympathetic dominance as HF approaches zero, as recently suggested (Billman, 2013). Two examples from the sample set reviewed demonstrate this potential volatility. Muralikrishnan et al. (2012) report a range of autonomic measures on Ishant Yoga practitioners vs. normal controls. At supine rest, the normal sample was described thus: *n* = 14, μ = 1.86, and *SD* = 6.35. As LF/HF ratio cannot be less than zero, this sample must contain one or more participants with a ratio of 15 or more, most likely due to HF power being minimal (a common occurrence when breathing rates are slow; see Saboul et al., 2014). As a consequence, the description of this sample by mean and standard deviation is unintelligible, as the distribution has profound positive skew.

Similarly, Chen et al. (2012) compared HRV metrics of resuscitated cardiac arrest patients, patients with sepsis and healthy controls. The raw LF and HF power of healthy controls ranged between approximately 12–100 times greater than all patient groups. For instance, post-cardiac arrest patients had both LF and HF spectral power of approximately 5 ms^2^, and healthy controls approximately 100 ms^2^. As a consequence, both of these groups had a median LF/HF ratio of 1. Alternatively, a difference was found between non-surviving (LF/HF = 0.2) and surviving (LF/HF = 3.1) cardiac arrest patients. However, none of the four spectral powers involved in this calculation had a median above 7.6 ms^2^.

None of the values above defined by ratio would be meaningful by themselves, and in the context of the original papers are appropriately reported and interpreted with both measures of the raw spectral power and total power. But as seen in Table 2, this is not the case for approximately 30% of published work.

### Limitations

There are several limitations to the present work, the most obvious of which is that it makes no attempt to propose a method by which spectral power *should* be assessed. There are a profound amount of variables to consider regarding such a question; whether spectral assumptions are appropriate in the first instance, which variant of spectral analysis is sufficient or optimal, how heartbeat series should be interpolated (if at all), how the series should be corrected (if at all) or windowed, and so forth.

On the same basis, this work records neither outcomes nor differences between free and paced breathing, specific time of day of recording, or participant age. The data set as reviewed is incapable of sustaining the scale of such a meta-analysis—for instance, Nunan et al. (2010) initially reviewed over 3000 individual pieces of research to draw a sample of *n* = 44 in which different methods of spectral analysis and the values they return within LF and HF bands could be compared, a requirement to meta-analytically compare regular measures of HRV spectral power to normalized or ratio variables. This relationship would almost certainly interact with the use of HRV to predict, investigate or stratify clinical conditions, as HRV values may be profoundly affected especially by autonomic and circulatory diseases. As a consequence, this work cannot speak to whether normalized or ratio units are capable of sustaining conclusions which are similar to those from raw values. Regardless of the basis on which they are characterized, or their internal consistency, or the manner of their usage, they may still reliably report the same or similar conclusions to other methods.

Finally, this work cannot determine the dispersion of values over the time of day or lifespan, or any relationship between these variables and the methodology used. This would be a worthy topic of future investigation, as HRV is used differentially within particular fields which are defined by time or age at recording (for instance, chronobiology or antenatal care), and the methodology between them is rarely compared.

## Conclusion

This review has concentrated on commonly used methodology, and hence the internal and external consistency, for collecting HRV by frequency analysis over the short term. In general, the nature of commonly used HRV metrics are not well understood, and these measurement are intimately related both on a mathematical level and in practice. Regardless of this, they are frequently treated as independent concepts and deployed redundantly. Additionally, insufficient attention is paid to the environment of data collection. None of these are trivial concerns; rather, they call into question the accuracy of the existing literature on HRV and warrant the re-establishment of an authoritative source for correct methodology and practice.

## Funding source

The author was supported by an Australian Postgraduate Award scholarship at the University of Sydney.

### Conflict of interest statement

The authors declare that the research was conducted in the absence of any commercial or financial relationships that could be construed as a potential conflict of interest.
